# Triage specific patient monitoring greatly impacts the number of alarming events in the Emergency Department

**DOI:** 10.1186/1757-7241-23-S1-A42

**Published:** 2015-07-16

**Authors:** Camilla LN Bech, Thomas Schmidt, Marianne Glud, Uffe K Wiil, Annmarie Lassen

**Affiliations:** 1 Department of Emergency Medicine, OUH Odense University Hospital, Odense C, Denmark; 2 The Mærsk Mc-Kinney Møller Institute, University of Southern Denmark, Odense M, Denmark

## Background

Electronic monitoring equipment usually have a predefined set of safe ranges and alarm thresholds that are manually altered depending on characteristics of patients and health care personnel. Preset alarm thresholds used to identify patients at risk of deterioration have poor specificity due to generalization of patient population, clinical needs, and care models. Staff at the Emergency Department is challenged by alarm fatigue which can ultimately be fatal; our aim with this study is to investigate the possible impact of triage specific thresholds on the number of generated alarms.

## Methods

Adult patients admitted to the bed units of the Emergency Department (ED) at Odense University Hospital (October 1^st ^2013-August 1^st ^2014) with vital signs measured on electronic monitors. Registration of personal identification number, timestamps, and all measured vital values, linked to triage information from the electronic logistic tool in the Department and stored locally in a research database. Level of severity defined by triage category given upon arrival and observation regimes according to the ADAPT triage model. We define an alarm event as three consecutive threshold violations. Alarm events for each patient were analyzed with 1) preset alarm thresholds configured in the patient monitors, and 2) individual thresholds as proposed by the ADAPT triage model.

## Results

884 triaged patients and a total of 628,839 vital sign measurements were registered. The frequency of alarm events generated was higher when preset alarm thresholds were used. 70 alarms opposed to 22 in the resuscitation category. 1,433 opposed to 450 in the very urgent category and 4,032 to 4,261 in the less urgent category. In the two less urgent triage categories; "not urgent" and "fast track", the number of generated alarms were distributed opposite with most generated alarm events with individualized thresholds; 5,661/1,844 and 144/12, respectively.

## Conclusion

The effect of individualized monitor alarm thresholds based on triage level has very different impact across groups. Given that patients are correctly triaged, the triage specific thresholds would have increased the total number of generated alarms by 35%. Primarily due to effect from patients triaged with the lowest levels of urgency.

